# Machine learning for prediction of histologic chorioamnionitis (stage ≥II) in parturients receiving labor analgesia: a retrospective multicentre cohort study

**DOI:** 10.3389/fmed.2026.1841139

**Published:** 2026-06-17

**Authors:** Chunping Li, Bo Liu, Liang Ling, Jianping Zhang, Jian Zhang, Fei Jia, Yong Tang

**Affiliations:** 1 Department of Anesthesiology, Sichuan Jinxin Xinan Women and Children's Hospital, Chengdu, Sichuan, China; 2 Department of Anesthesiology, Chengdu Jinjiang District Women and Children Health Hospital, Chengdu, Sichuan, China; 3 Department of Anesthesiology, Chongqing General Hospital, Chongqing University, Chongqing, China; 4 Department of Anesthesiology, Sichuan Women's and Children's Hospital/Women's and Children's Hospital, Chengdu Medical College, Chengdu, Sichuan, China

**Keywords:** histologic chorioamnionitis, labor analgesia, machine learning, predictive model, shap

## Abstract

**Background:**

Histological chorioamnionitis (HCA) is a serious pregnancy complication, but early diagnosis is challenging, especially in parturients receiving labor analgesia, where identification is even more difficult. Therefore, we developed and validated a machine learning model for early prediction of HCA (stage ≥II) intended for use at the time of epidural-related maternal fever (ERMF) onset, before delivery and confirmatory histopathology.

**Methods:**

This study utilized a multicenter retrospective cohort dataset, including parturients receiving labor analgesia and completed placental pathological examination. Candidate features were extracted from electronic health records (EHR), followed by data preprocessing procedures such as addressing multicollinearity, Z-score standardization, and minority class weighting. Subsequently, three machine learning models—logistic regression (LR), random forest (RF), and extreme gradient boosting (XGBoost)—were developed and compared, with their performance evaluated through internal and independent external validation. Finally, the SHapley Additive exPlanations (SHAP) method was used to interpret the best-performing model, aiming to clarify the predictive contribution of each feature.

**Results:**

A total of 2,715 parturients were included in this study, of which 676 cases (24.9%) were diagnosed with HCA (stage ≥II). After feature selection, the model retained 6 key features. The RF model exhibited the best performance, achieving an Area Under the Curve(AUC) of 0.945 in the internal validation set and an AUC of 0.849 in the independent external validation set, demonstrating balanced sensitivity (0.957) and specificity (0.867). SHAP analysis indicated that body mass index (BMI) was the most important predictive factor.

**Conclusions:**

The RF model performed the best in predicting the risk of HCA (stage ≥II) in parturients receiving labor analgesia. SHAP analysis further revealed that BMI was the most important predictive factor in the model.

## 1 Introduction

Histological chorioamnionitis (HCA) is a pathological condition characterized by an inflammatory response during pregnancy, manifested by the infiltration of neutrophils observed in pathological examinations (1, 2). HCA can lead to severe adverse pregnancy outcomes such as uterine infection, premature rupture of membranes, placental abruption, and neonatal pneumonia (3–5). HCA (Stage ≥II) represents a more advanced and clinically significant pathological state, characterized by neutrophil infiltration extending into the chorionic stroma and/or amnion, and is strongly associated with adverse maternal and neonatal outcomes. However, early diagnosis of HCA is challenging, as it can typically only be confirmed through placental pathological examination after delivery. This poses significant challenges for timely diagnosis of HCA (6). Particularly in parturients receiving neuraxial labor analgesia, labor analgesia may lead to Epidural-Related Maternal Fever (ERMF), which is often a major manifestation of HCA, further complicating the identification of HCA (7, 8).

The application of machine learning models to EHR system data holds significant potential for the diagnosis of HCA (9–11). In this context, EHR data are particularly valuable because they comprehensively document laboratory test results, current symptoms, maternal comorbidities, and other relevant information. However, clinicians often face challenges in utilizing this information efficiently. Machine learning models are capable of analyzing large volumes of complex data, identifying patterns and associations that may be imperceptible to humans, thereby uncovering potential disease progression trends and diagnostic clues. This enables machine learning models to provide valuable insights when processing vast amounts of medical data, assisting in clinical decision-making and identifying progression patterns of HCA (12, 13).

This study aimed to investigate whether a machine learning model trained on EHR-based clinical data could predict the risk of developing HCA (stage ≥II) in parturients receiving neuraxial labor analgesia, thereby improving prognosis and delivery trajectories. Building upon our previous work (14), this research advances the field in two key aspects: by employing a machine learning prediction model and by incorporating independent external validation.

## 2 Methods

### 2.1 Study design and participants

This retrospective multicentre cohort study was approved by the ethics committees of all participating centers: Chengdu Jinjiang District Women and Child Health Hospital [approval number: 2024 trial (20)], Sichuan Women's and Children's Hospital (approval number: 20230807-216), and Sichuan Jinxin Xinan Women and Children's Hospital [approval number: 2024 trial (2)]. All research was performed in accordance with the Declaration of Helsinki and relevant guidelines and regulations. Written informed consent was waived by the ethics committees due to the retrospective nature of the study, which involved the analysis of anonymized electronic health records. The reporting of data handling and analysis follows the TRIPOD (Transparent Reporting of a Multivariable Prediction Model for Individual Prognosis or Diagnosis) guideline (15).

### 2.2 Parturients

**Figure 1:**
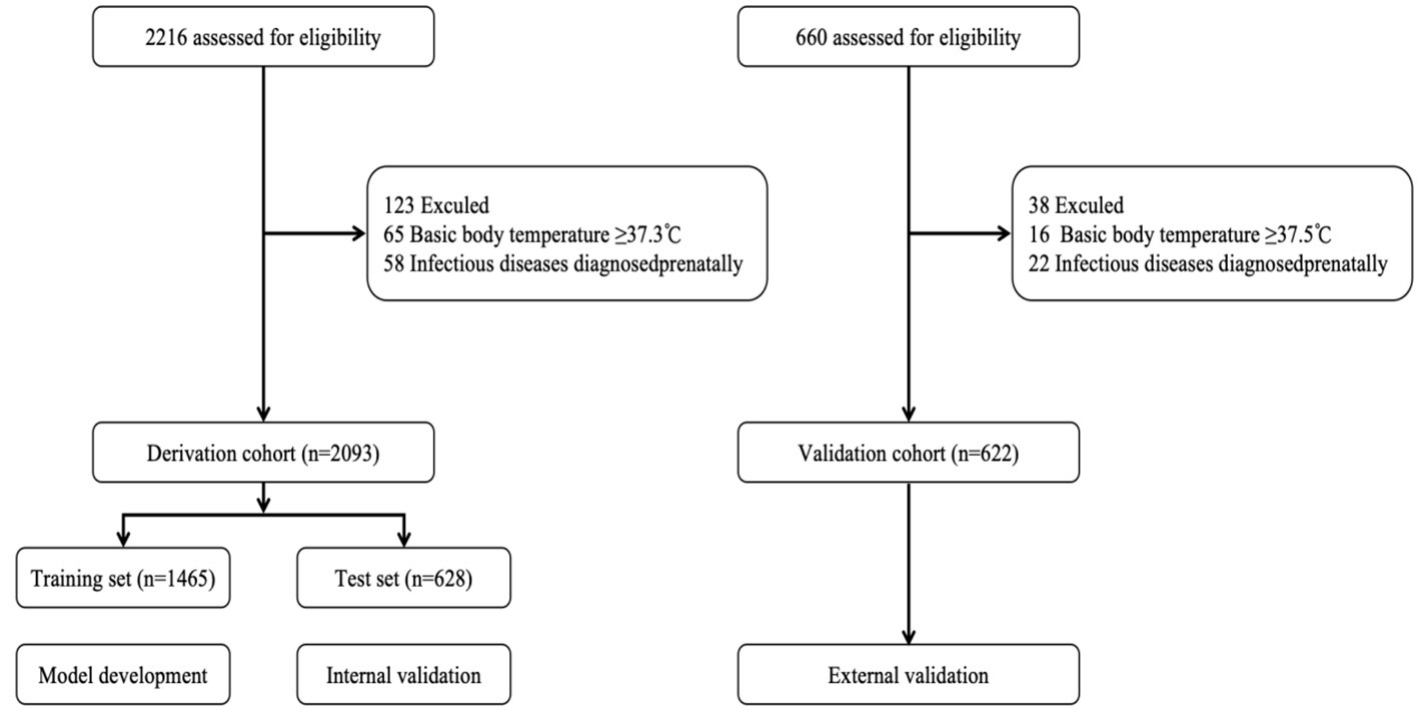
Participants screening flow chart.

This study included parturients admitted between January 1, 2019, and December 31, 2024, from two derivation hospitals (Chengdu Jinjiang District Women and Child Health Hospital and Sichuan Women's and Children's Hospital) and one external validation hospital (Sichuan Jinxin Xinan Women and Children's Hospital). The participant screening process is illustrated in Figure 1. Inclusion criteria were: (1) voluntary receipt of neuraxial labor analgesia; (2) American Society of Anesthesiologists (ASA) physical status class II; (3) singleton pregnancy; (4) availability of placental histopathological examination after delivery. Exclusion criteria included: (1) a baseline body temperature ≥ 37.3 °C prior to the administration of labor analgesia; (2) documented evidence of other clear sources of infection; and (3) incomplete or missing records.

### 2.3 Data split

The data were partitioned into a derivation cohort and an independent external validation cohort by hospital site. Specifically, parturients from Chengdu Jinjiang District Women and Child Health Hospital and Sichuan Women's and Children's Hospital constituted the derivation cohort, which was used for model development and internal validation, parturients from Sichuan Jinxin Xinan Women and Children's Hospital formed the external validation cohort for testing the model's generalizability (Figure 1).

### 2.4 Primary outcome definition

The primary outcome was HCA (stage ≥II). After delivery, a standardized placental tissue sample (2 cm × 2 cm) extending from the rupture site to the placental margin was collected for histopathological examination. All specimens were evaluated by experienced pathologists blinded to clinical information. Diagnosis of HCA was established when ≥5 neutrophils per high-power field were observed diffusely infiltrating the chorionic plate and/or amnion (16). According to established pathological staging criteria, HCA was classified into three stages: stage I (early, acute subchorionitis/chorioamnionitis), stage II (acute chorioamnionitis with neutrophil extension into the chorionic stroma and/or amnion), and stage III (necrotizing chorioamnionitis characterized by karyorrhexis, amnion epithelial necrosis, and/or hyalinization of the amnion basement membrane). For this study, only stages II and III were considered as positive outcomes.

### 2.5 Data collection and processing

Data were extracted from the EHRs of the participating centers. The collected variables encompassed demographic information, obstetric characteristics, maternal comorbidities, intrapartum clinical and laboratory findings, as well as neonatal characteristics. Specifically, we recorded the estimated neonatal weight along with maternal age, BMI, primiparity (yes/no), gestational age, and the occurrence of premature rupture of membranes (PROM). Placental weight and the presence of meconium-stained amniotic fluid were recorded. Maternal comorbidities included gestational diabetes mellitus (GDM), hypertension, anemia, hepatitis B, and hypothyroidism. The baseline body temperature and the maximum maternal body temperature during labor was documented. Relevant laboratory parameters during the ERMF period comprised white blood cell count (WBC), red blood cell count (RBC), hemoglobin concentration (Hb), platelet count (PLT), neutrophil count (NEUT), lymphocyte count (LYM), monocyte count (Mono), the derived ratios of neutrophils-to-lymphocytes (NLR), monocytes-to-lymphocytes (MLR), platelets-to-lymphocytes (PLR), and monocyte percentage (M%). The CRP level was also collected. The primary outcome was based on the histopathological examination of the placenta, diagnosing HCA (stage ≥II).

A standardized preprocessing pipeline was applied to ensure data quality. As participants with incomplete records were excluded during screening, no imputation for missing values was performed. To mitigate multicollinearity, pairwise Pearson correlation coefficients were calculated for continuous variables. Feature pairs with correlations >0.6 were identified as collinear. For each such pair, we compared the point-biserial correlations of both variables with the binary outcome (HCA stage ≥II) and removed the variable with the lower absolute correlation. All preprocessing parameters were derived solely from the training set and then applied to the internal validation set and the external validation cohort. No information from the validation data was used during preprocessing

### 2.6 Feature selection and dimensionality reduction

To further identify key predictive variables and control model complexity, we performed feature selection using Least Absolute Shrinkage and Selection Operator (LASSO) regression after addressing multicollinearity (17). Selection was based on ten-fold cross-validation to achieve a more streamlined set of variables while ensuring predictive performance. LASSO feature selection was performed on the training set only. The selected features were then fixed and used as the input variables for all subsequent model development and validation steps, without re-selection or adjustment based on the internal validation set or external validation cohort.

### 2.7 Model development, comparison, and selection

Considering that the prevalence of HCA (stage ≥II) in the training set was 23.7%, we assigned higher weights to the minority class during model training to address the class imbalance problem (18). The derivation cohort was randomly split into a training set and an internal validation set in a 7:3 ratio (19). On the training set, we trained three machine learning algorithms: LR, RF, and XGBoost. Hyperparameters for all models were optimized using Bayesian optimization via 10-fold cross-validation (20). The search ranges and final selected values for each model are reported in Supplementary Table 1. All hyperparameter tuning was performed on the training set only, with no information from the validation sets used during model development.

On the internal validation set, we systematically evaluated and compared the performance of each model in terms of both discriminative ability and classification accuracy. The specific assessment framework was as follows: (1) The AUC and the area under the precision-recall curve (AUPRC) were adopted as the core metrics for evaluating the model's discriminative ability; (2) A comprehensive examination of classification accuracy metrics was conducted, including sensitivity, specificity, precision, balanced accuracy, and the F1 score. (3) Model calibration was assessed using the Brier score. Based on the results of this multi-dimensional evaluation, the model demonstrating the best overall performance on the internal validation set was selected as the final candidate model. Subsequently, the selected candidate model was subjected to final testing on the completely independent external validation cohort to independently assess its generalizability.

The model is designed for application at ERMF onset, when all required predictors are already available, enabling early HCA (stage ≥II) risk assessment before delivery and histopathology.

### 2.8 Model interpretation

To enhance the clinical interpretability of the model, this study employed the SHAP method to interpret the final model. The SHAP summary plot identified and ranked the key features for predicting HCA (stage ≥II) at a global level; the SHAP dependence plots revealed the specific associations between these features and the predicted risk; and the SHAP force plots demonstrated how the prediction for an individual parturient was driven by her specific feature values. This interpretability framework makes the model's decision-making transparent, thereby supporting individualized clinical assessment in practice.

### 2.9 Sample size

We utilized the “pmsampsize” R package (version 4.3.1; R Development Core Team, Vienna, Austria) to calculate the required sample size. This calculation was based on a c-statistic of 0.744, 27 candidate parameters, and a prevalence rate of 30.0%, following the formula proposed by Riley and colleagues (14, 21). Taking into account a shrinkage of 0.9, the minimum sample size was determined to be 1,464. The training set within the derivation cohort consisted of 1,465 participants, meeting the predefined criteria.

### 2.10 Statistical analysis

The Kolmogorov-Smirnov test was used to evaluate the normal distribution of continuous variables. For normally distributed continuous variables, data are presented as the mean ± standard deviation (SD) and were analyzed via the independent-sample t-test. Nonnormally distributed continuous variables are expressed as the median (M) and interquartile range (IQR) and were compared using the Mann-Whitney U-test. Categorical variables are presented as numbers and percentages (%) and compared with the Chi–square test or Fisher's exact test as appropriate. A *P*-value of less than 0.05 was considered statistically significant. The model development and validation were performed using R version 4.3.1 and SPSS software version 25.0.

## 3 Results

A total of 2,715 parturients were included in this study, and 676 of them (24.9%) were identified as having HCA (stage ≥II). Within the derivation cohort of 2,093 participants, 495 (23.7%) were classified as HCA (stage ≥II), while in the validation cohort of 622 participants, 181 (29.1%) met the same diagnostic criteria. The comparison of characteristics between the training set and the test set is presented in Table 1. The proportion of hypothyroidism was significantly higher in the training set than in the test set (13.4% vs. 8.9%, *P* < 0.05). No other variables showed statistically significant differences between the two sets.

**Table 1:** Characteristics comparison between training set and test set.

Characteristics	Overall	Training set	Test set	*P* value
	(*n* = 2093)	(*n* = 1465)	(*n* = 628)	
Patient characteristics				
Age (years)	29 (27–31)	29 (27–31)	28 (27–31)	0.115
BMI (kg/m^2^)	25.1 (22.4–27.3)	25.1 (22.4–27.4)	25.2 (22.4–27.3)	0.630
Gestational age (week)	39.9 (39.0–40.4)	39.9 (39.0–40.4)	39.9 (39.0–40.4)	0.569
Basal temperature (°C)	36.5 (36.4–36.6)	36.5 (36.4–36.6)	36.5 (36.4)36.6)	0.371
Maximum temperature (°C)	38.3 (38.0–38.5)	38.3 (38.0–38.5)	38.2 (38.0–38.5)	0.839
Estimated neonatal weight (g)	3350 (3140–3578)	3350 (3150–3550)	3358.5 ± 369.3	0.731
Placental weight (g)	560 (500–645)	505 (500–640)	560 (500–650)	0.679
Primiparity (%)	1987 (94.9)	1388 (94.7)	599 (95.4)	0.588
PROM (%)	669 (31.9)	452 (30.9)	217 (34.6)	0.102
Meconium-stained amniotic fluid (%)	550 (26.2)	381 (26.0)	169 (26.9)	0.665
Comorbidity				
GDM (%)	498 (23.7)	351 (24.0)	147 (23.4)	0.823
Hypertension (%)	122 (5.8)	93 (6.3)	29 (4.6)	0.128
Anemia (%)	733 (35.0)	503 (34.3)	230 (36.6)	0.318
Hepatitis B (%)	106 (5.0)	71 (4.8)	35 (5.6)	0.514
Hypothyroidism (%)	253 (12.0)	197 (13.4)	56 (8.9)	< 0.05
Laboratory tests				
WBC (10^9^/L)	15.0 (13.0–17.3)	15.0 (12.9–17.3)	15.1 (13.1–17.5)	0.233
RBC (10^12^/L)	4.0 (3.7–4.2)	4.0 (3.7–4.2)	4.0 ± 0.4	0.885
Hb (g/L)	122 (114–131)	122 (114–130)	122 (114–131)	0.909
NEUT (10^9^/L)	13.2 (11.1–15.2)	13.1 (11.0–15.2)	13.3 (11.2–15.3)	0.243
LYM (10^9^/L)	1.0 (0.8–1.3)	1.0 (0.8–1.3)	1.0 (0.8–1.3)	0.810
PLT (10^9^/L)	156 (127–190)	156 (125–188)	157 (127–193)	0.541
CRP (mg/L)	17.8 (10.1–34.3)	17.4 (10.0–33.1)	18.9 (10.2–35.8)	0.208
Mono (10^9^/L)	0.8 (0.6–1.0)	0.8 (0.6–1.0)	0.8 (0.6–1.0)	0.112
NLR	12.7 (10.0–16.3)	12.7 (10.0–16.3)	12.7 (10.0–16.1)	0.994
MLR	0.8 (0.6–1.0)	0.8 (0.6–1.0)	0.8 (0.6–1.0)	0.413
PLR	156.5 (115.6–201.9)	155.7 (115.6–201.1)	157.0 (115.6–203.5)	0.546
M%	0.05 (0.04–0.06)	0.05 (0.04–0.06)	0.05 (0.04–0.06)	0.472

BMI, body mass index; PROM, premature rupture of membranes; GDM, gestational diabetes mellitus; WBC, white blood cell count; RBC, red blood cell count; Hb, hemoglobin; NEUT, neutrophil count; LYM, lymphocyte count; PLT, platelet count; CRP, C-reactive protein; Mono, monocyte count; NLR, neutrophil-to-lymphocyte ratio; MLR, monocyte-to-lymphocyte ratio; PLR, platelet-to-lymphocyte ratio; M%, monocyte percentage.

### 3.1 Feature selection

**Figure 2:**
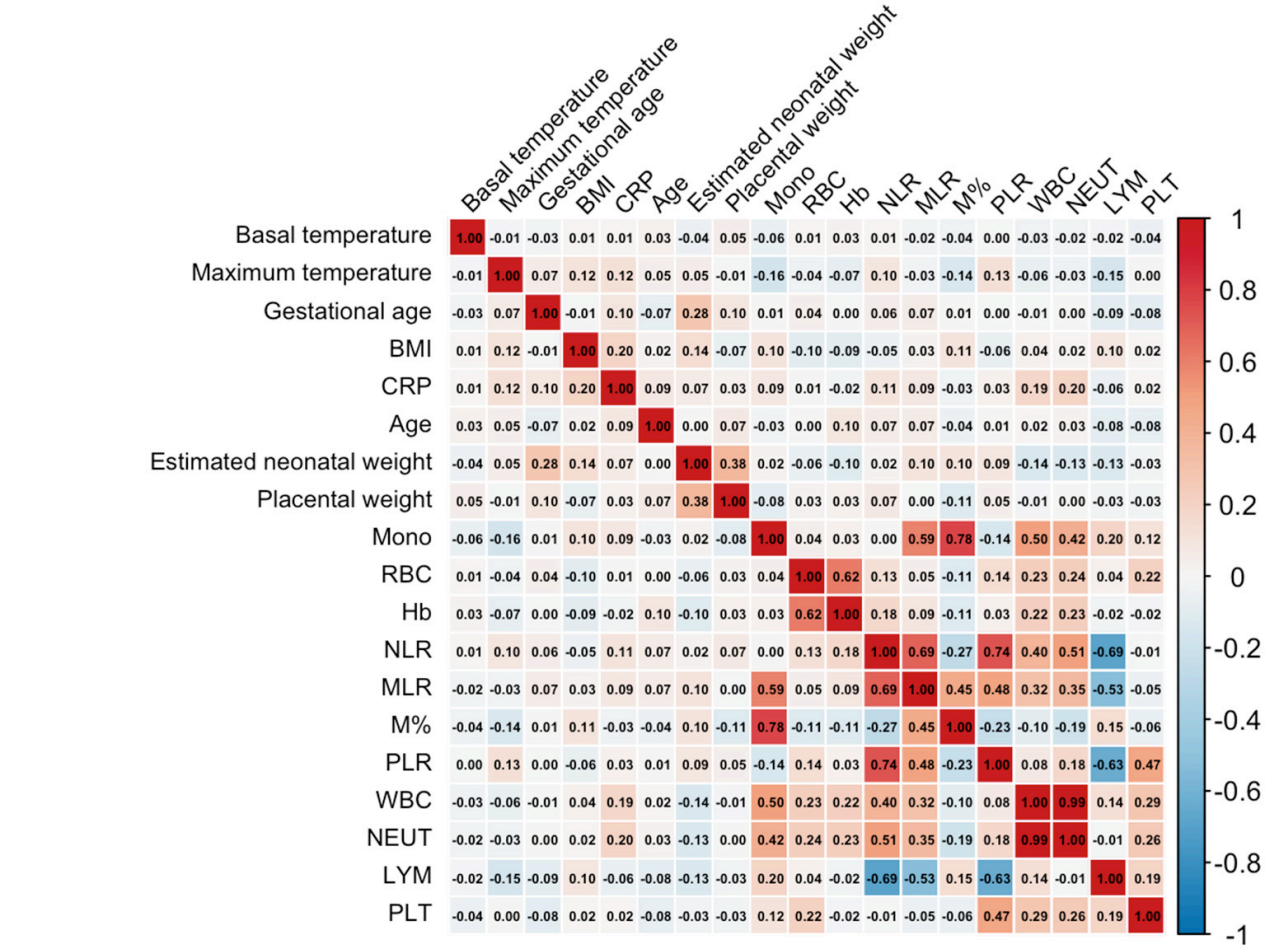
Correlation matrix for assessing multicollinearity among candidate continuous variables.

Before constructing the machine learning model, we performed feature selection on continuous variables to address multicollinearity. The correlation analysis (Figure 2) revealed seven pairs of variables with correlation coefficients >0.6 (Supplementary Table 2). For each correlated pair, we compared their point-biserial correlation coefficients with the outcome (HCA stage ≥II) and retained the variable with the stronger association. Based on this criterion, NEUT, Mono, MLR, LYM, and Hb were ultimately included (Supplementary Table 3). After excluding highly correlated features, six features were finally selected via LASSO regression (see Supplementary Figure 1): maximum temperature, Gestational age, BMI, CRP, Meconium-stained amniotic fluid, and PLT.

### 3.2 Model performance

**Figure 3:**
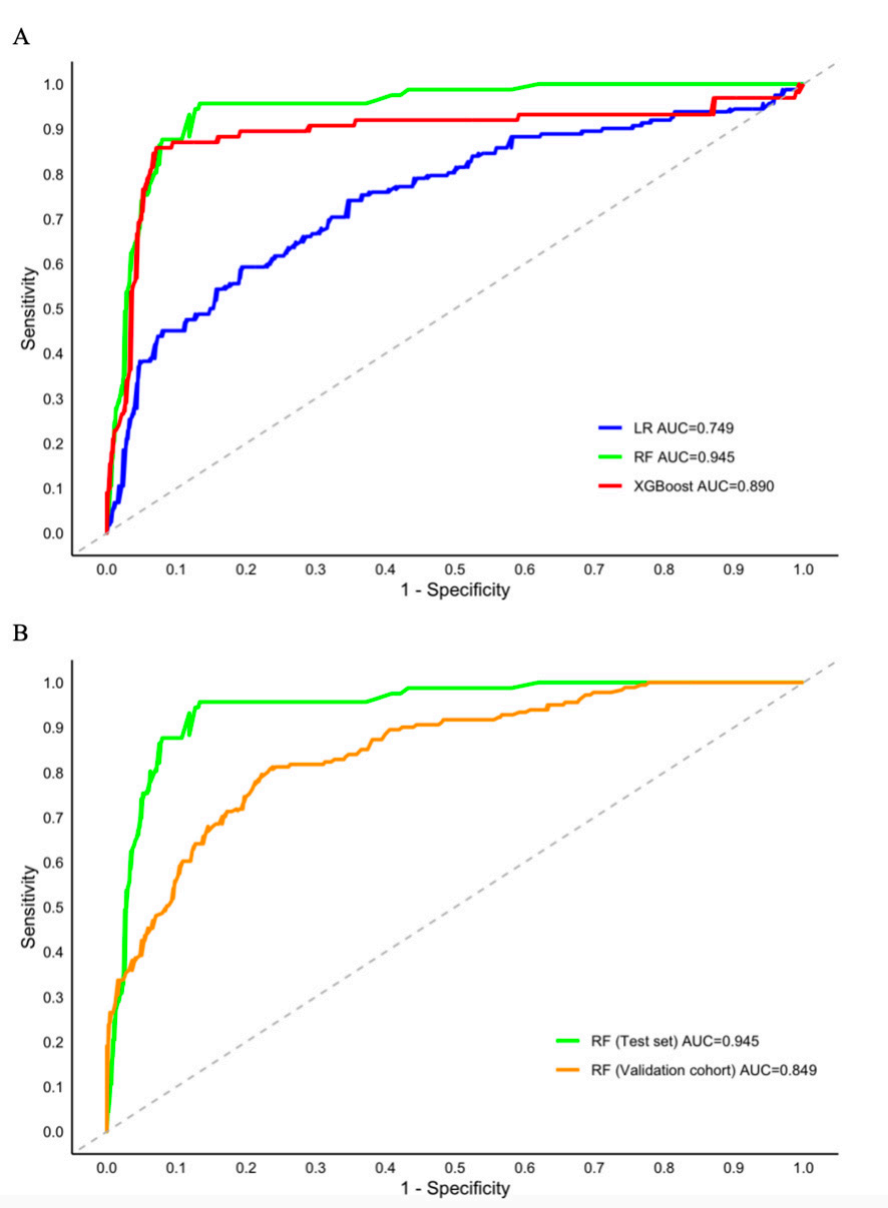
ROC curves. **(A)** ROC curves of the three prediction models on the test set. **(B)** ROC curve of the selected RF model on the independent external validation cohort.

The RF model demonstrated the best performance across multiple evaluation metrics (see Table 2): it achieved the highest values in AUC (0.945, with the ROC curve for the internal validation set shown in Figure 3A), sensitivity (0.957), balanced accuracy (0.912), and F1 score (0.818), while also maintaining high specificity (0.867) and precision (0.714). Therefore, based on a comprehensive consideration of discriminative ability, classification balance, and clinical applicability, the RF model was selected as the optimal predictive model. When applied to the independent external validation cohort, the model achieved an AUC of 0.849 (ROC curve shown in Figure 3B), further confirming its favorable generalizability. Additional performance metrics for the external validation cohort are presented in Table 2. The Brier scores for LR, RF, and XGBoost were 0.160, 0.081, and 0.089, respectively (Supplementary Table 4), further confirming the superior calibration of the RF model.

**Table 2:** Performance evaluation of machine learning models.

Machine learner	AUC	AUPRC	Sensitivity	Specificity	Precision	Balanced accuracy	F1 score
**LR (test set)**	0.749 (0.702–0.796)	0.544	0.889	0.363	0.327	0.626	0.478
**RF (test set)**	0.945 (0.926–0.964)	0.828	0.957	0.867	0.714	0.912	0.818
**XGBoost (test set)**	0.890 (0.852–0.928)	0.792	0.858	0.914	0.777	0.886	0.815
**RF (external validation cohort)**	0.849 (0.815–0.882)	0.739	0.812	0.762	0.583	0.787	0.679

LR, logistic regression; RF, random forest; XGBoost, extreme gradient boosting.

### 3.3 Clinical validation

In the independent external validation cohort, the decision curve of the RF model (Supplementary Figure 2) showed a positive clinical net benefit over a wide range of threshold probabilities, while the calibration curve (Supplementary Figure 3) demonstrated good agreement between predicted probabilities and observed outcomes.

### 3.4 SHAP analysis

**Figure 4:**
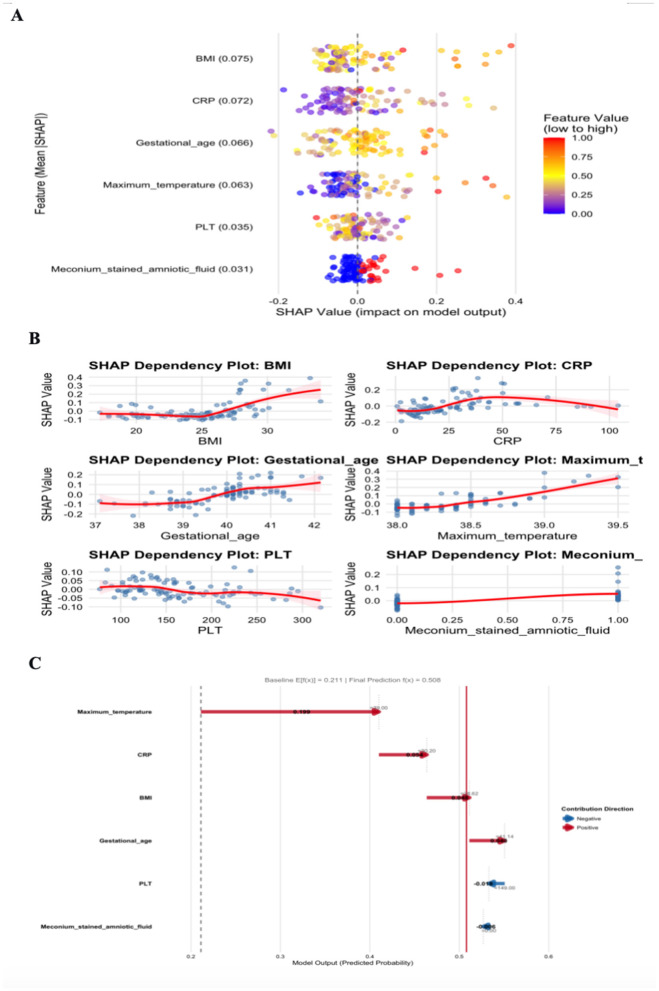
SHAP plots. **(A)** SHAP summary plot. **(B)** SHAP dependence plots. **(C)** SHAP waterfall plot illustrating individualized prediction of HCA (stage ≥II) risk.

Based on the feature importance ranking derived from the SHAP summary plot (Figure 4A), BMI was identified as the most influential predictor for HCA (stage ≥II), with a mean absolute SHAP value of 0.075. This was followed by CRP (0.072), gestational age (0.066), maximum maternal temperature (0.063), PLT (0.035), and meconium-stained amniotic fluid (0.031). The detailed relationships between each feature and the model output are further visualized in the corresponding SHAP dependence plots (Figure 4B). To better illustrate the individual influence of each variable, the SHAP waterfall plot depicting the personalized risk prediction for HCA (stage ≥II) in a representative case is presented in Figure 4C.

### 3.5 Sensitivity analysis

Sensitivity analysis results showed that after sequentially excluding each of the six key variables in the test set, the model's AUC remained within the range of 0.931–0.949, indicating good robustness of the model. However, in the independent external validation cohort, the model performance significantly declined after excluding certain variables—for example, the AUC dropped to 0.737 after excluding gestational age (Supplementary Table 5).

## 4 Discussion

Based on multicenter electronic medical record data, this study employed an interpretable machine learning approach to develop a prediction model incorporating only six clinical indicators for assessing the risk of HCA (stage ≥II) in parturients receiving neuraxial labor analgesia. Among the three machine learning models compared, the RF model demonstrated the best performance. Using the SHAP method for feature interpretation, the results revealed that BMI and CRP were the most influential predictors.

The key advantage of our model lies in its reliance on only six routine clinical indicators for prediction, without depending on subjective assessments. To the best of our knowledge, this is the first machine learning-based study specifically designed to predict the risk of HCA (stage ≥II) in parturients receiving labor analgesia. The model is intended for application at the onset of ERMF, before placental pathology is available. It thus enables risk assessment prior to delivery, facilitating early identification of high-risk parturients and thereby avoiding delays in intervention due to waiting for pathological results. Furthermore, its automated risk stratification mechanism effectively reduces potential bias and inconsistency associated with human assessment.

In our study, three models—LR, RF, and XGBoost—were selected for development and comparison. LR was used as a performance benchmark due to its strong interpretability (22). RF and XGBoost were included for their ability to automatically model complex nonlinear relationships (23, 24). Compared with traditional LR, these machine learning approaches offer distinct advantages: they can automatically identify important features, reducing subjective bias; enhance generalizability through ensemble strategies, thereby mitigating overfitting; and flexibly adapt to diverse types of clinical data without strict assumptions on data distribution.

The outstanding performance of the RF model in this study can be attributed to several key factors: it effectively handles complex nonlinear relationships in clinical data, accommodates mixed-type features well, enhances generalization through ensemble learning, and maintains both high predictive performance and good interpretability. In addition, several measures were taken to minimize overfitting and ensure model robustness. Bayesian optimization with ten-fold cross-validation was used for hyperparameter tuning, class weighting was applied to address outcome imbalance, and the final feature set was limited to six variables via LASSO regression. The stability of the model was further supported by sensitivity analysis, in which AUC remained stable after sequentially removing each feature. These characteristics make RF particularly suitable for HCA risk prediction tasks based on multicenter clinical data (25). RF has demonstrated reliable performance in numerous disease prediction studies using EHR data. Recently, in a study predicting in-hospital mortality for patients with arterial ischemic stroke in the intensive care unit, the RF model outperformed other machine learning approaches, and its predictions were clearly explained through SHAP analysis (26). This further confirms the model's dual advantages of strong performance and interpretability in medical prediction tasks.

To enhance the clinical comprehensibility and decision transparency of the model, this study employed the SHAP method to interpret the RF model. SHAP, as a robust feature attribution framework, provides consistent and reliable interpretability for machine learning models, aiding in transforming prediction results into actionable clinical insights (27). Through SHAP summary plots and dependence plots, we were able to globally assess feature importance and conduct in-depth analyses of the nonlinear relationships between specific features and predicted risk. SHAP analysis clarified the relative importance of each feature, with the most influential being BMI, CRP, gestational age, maximum maternal temperature, PLT, and meconium-stained amniotic fluid, in descending order. The identification of these key indicators renders the model's decision-making process transparent, thereby enhancing clinical understanding of the primary drivers behind the risk prediction.

The machine learning model developed in this study ultimately identified six predictive variables. Compared with our team's earlier prediction model based on LR, the two studies show high consistency in five key variables: BMI, CRP, gestational age, maximum maternal temperature, and meconium-stained amniotic fluid (14). This convergence, on the one hand, confirms the stable and reproducible association of these indicators—serving as core biomarkers of inflammation, metabolism, and pregnancy status—in predicting the risk of HCA (stage≥II). Notably, among these five consistent variables, BMI emerged as the most important predictor in our current machine learning model. As we previously reported, obese parturients may have chronic inflammatory responses that become activated during labor, and their adipose tissue may produce more inflammatory factors that participate in temperature regulation (14). This pro-inflammatory state likely lowers the threshold for intra-amniotic inflammation and increases HCA risk, which may explain why BMI ranked first in the machine learning model.

It is noteworthy that the present model additionally incorporated PLT as an important protective predictor. SHAP analysis revealed that higher PLT values were significantly associated with a reduced risk of HCA (stage≥II). PLT not only participate in coagulation but also function as active immune regulators, with their functional state being highly dependent on finely tuned lipid metabolism. Through the release of signaling molecules such as sphingolipids and oxidized lipids, platelets modulate local inflammatory responses in a paracrine manner and can effectively induce macrophages to adopt a pro-resolving phenotype, enhancing their capacity to clear apoptotic cells and thereby exerting anti-inflammatory effects. This process does not rely on conventional M2 polarization mechanisms but is instead mediated via prostaglandin E2-activated EP4 receptors (28–30).

This study has significant implications for clinical practice. First, our model demonstrates good predictive performance for HCA (stage ≥II) and can be used for non-invasive risk assessment based on maternal indicators, supporting early warning efforts. Second, the study identified modifiable predictive factors such as BMI, CRP, and highest body temperature, suggesting that optimizing maternal metabolic status and monitoring inflammatory markers may help reduce the risk of severe HCA. Third, As a preliminary decision-support suggestion, a predicted probability above approximately 50% (i.e., a relative risk exceeding 2-fold) for HCA (stage ≥II) might warrant consideration of intensified fetal monitoring, early neonatal team notification, and timely intrapartum antibiotics. Expedited delivery could be considered when HCA is strongly suspected, but this threshold should be interpreted cautiously and validated in future prospective studies. Lastly, although the diagnosis of HCA relies on postpartum placental pathology, the machine learning model developed in this study can serve as a real-time risk assessment tool, promoting the shift of obstetric care toward a more proactive prevention approach. Additionally, it provides new insights into the role of PLT in immune regulation during intrauterine inflammation.

This study has certain limitations. First, the retrospective electronic medical record data may be incomplete or lack uniform standardization. Second, the samples were sourced from three specialized hospitals in the same region, so caution should be exercised when extrapolating the conclusions to other populations. Third, potential confounding factors such as the use of antenatal antibiotics were not included in the model. Fourth, this model was developed specifically for parturients with ERMF, and its applicability to afebrile parturients remains unevaluated. Fifth, we did not calculate calibration intercept or slope, which are recommended for clinical prediction models. Future studies should include these metrics. Finally, further prospective multicenter studies are warranted to validate the model's practical utility.

## 5 Conclusions

This study successfully developed and validated machine learning models based on LR, RF, and XGBoost to predict the risk of HCA (stage ≥II) in parturients receiving labor analgesia. The comparison of the models showed that the RF model performed best in terms of discrimination and predictive accuracy. SHAP analysis further provided explanations for the model decisions at both global and individual levels, identifying BMI as the most important predictive factor.

## Data Availability

The raw data supporting the conclusions of this article will be made available by the authors, without undue reservation.
