# Supplementary material for: Machine learning for prediction of histologic chorioamnionitis (stage ≥II) in parturients receiving labor analgesia: a retrospective multicentre cohort study
Source: Front Med (Lausanne). 2026 Jun 17;13:1841139. doi: 10.3389/fmed.2026.1841139 (PMC13318988; doi:10.3389/fmed.2026.1841139)
Supplement: Supplementary file 1 [file Image_1.pdf]

A

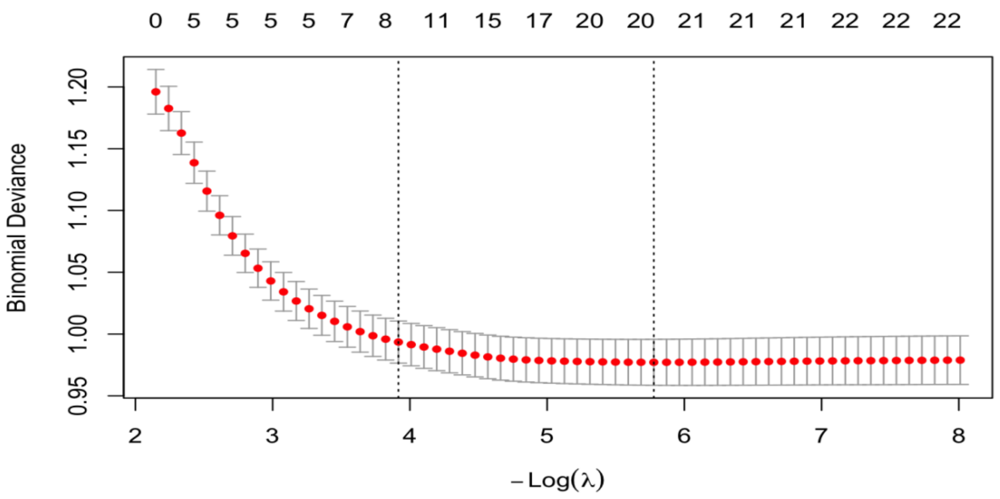

B

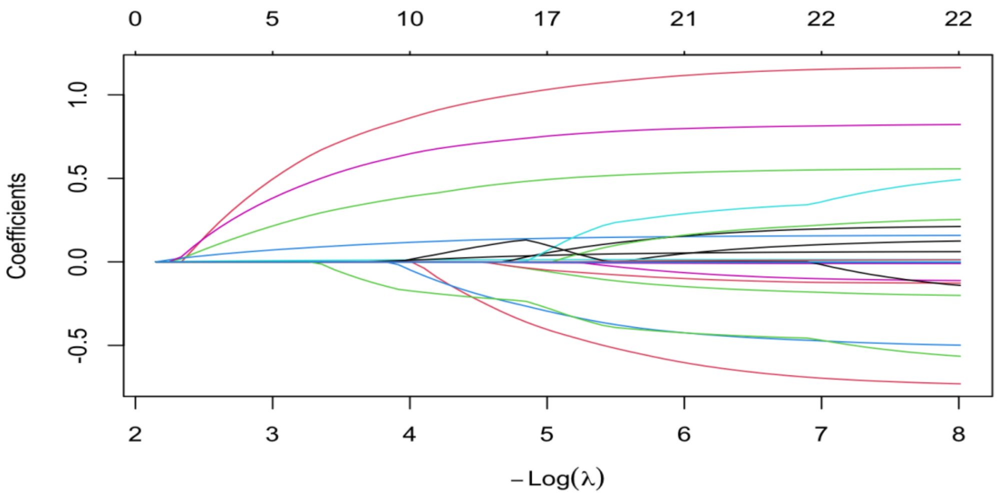

Supplementary Figure 1. LASSO regression for predicting HCA (stage ≥ II). LASSO regression uses a penalty function controlled by the hyperparameter λ to shrink coefficients and select key variables. Through ten-fold cross-validation, we identified the optimal λ (lambda 1se), which yields a parsimonious model with good predictive performance (Supplementary Figure 1A). Supplementary Figure 1B shows the coefficient paths of 22 features associated with HCA (stage ≥ II), illustrating how increasing penalty strength promotes variable sparsity. Ultimately, six key predictors were selected: Maximum temperature, Gestational age, BMI, CRP, Meconium-stained amniotic fluid, and PLT.
