# Supplementary material for: Machine learning for prediction of histologic chorioamnionitis (stage ≥II) in parturients receiving labor analgesia: a retrospective multicentre cohort study
Source: Front Med (Lausanne). 2026 Jun 17;13:1841139. doi: 10.3389/fmed.2026.1841139 (PMC13318988; doi:10.3389/fmed.2026.1841139)
Supplement: Supplementary file 2 [file Image_2.pdf]

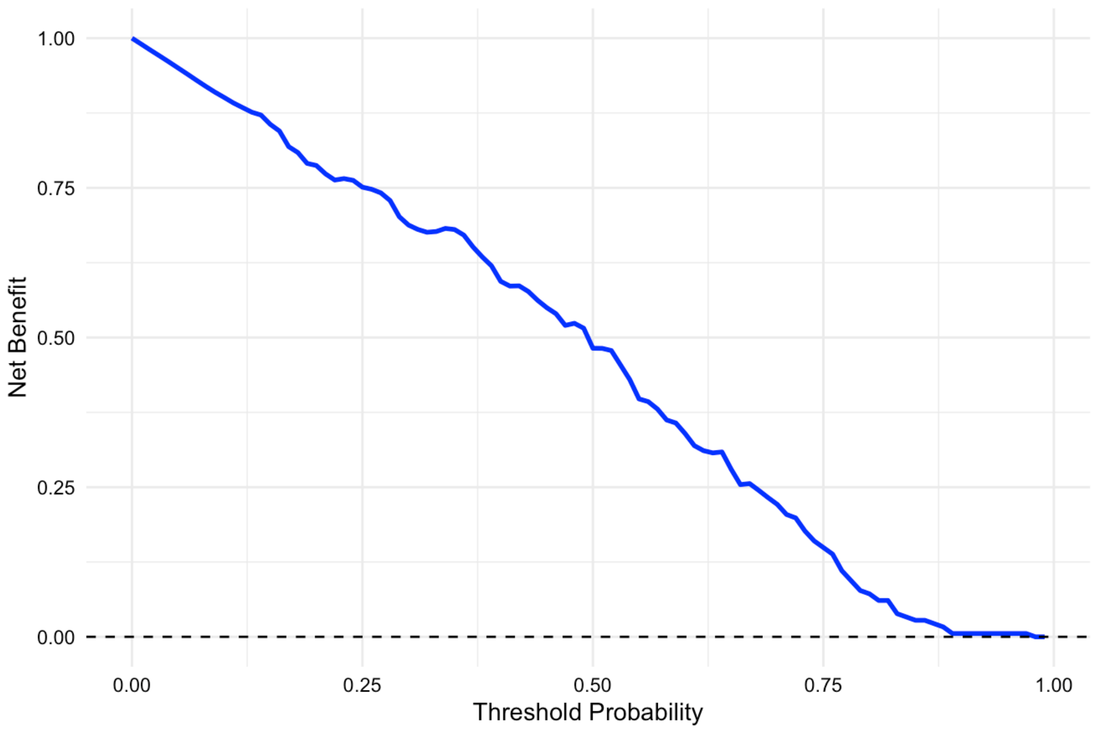

Supplementary Figure 2. Decision Curve Analysis of the RF Model in the External Validation Cohort.

The Decision Curve Analysis illustrates the net benefit of using the RF model across a range of threshold probabilities in the independent external validation cohort. The blue solid curve represents the net benefit of the model, while the grey solid horizontal line indicates the "treat none" strategy (net benefit = 0). The curve demonstrates that the model provides positive net clinical benefit across a wide threshold probability range (from 0.02 to 1.00), supporting its utility for clinical decision-making regarding HCA (stage ≥ II) risk in parturients receiving labor analgesia.
