# Supplementary material for: Machine learning for prediction of histologic chorioamnionitis (stage ≥II) in parturients receiving labor analgesia: a retrospective multicentre cohort study
Source: Front Med (Lausanne). 2026 Jun 17;13:1841139. doi: 10.3389/fmed.2026.1841139 (PMC13318988; doi:10.3389/fmed.2026.1841139)
Supplement: Supplementary file 3 [file Image_3.pdf]

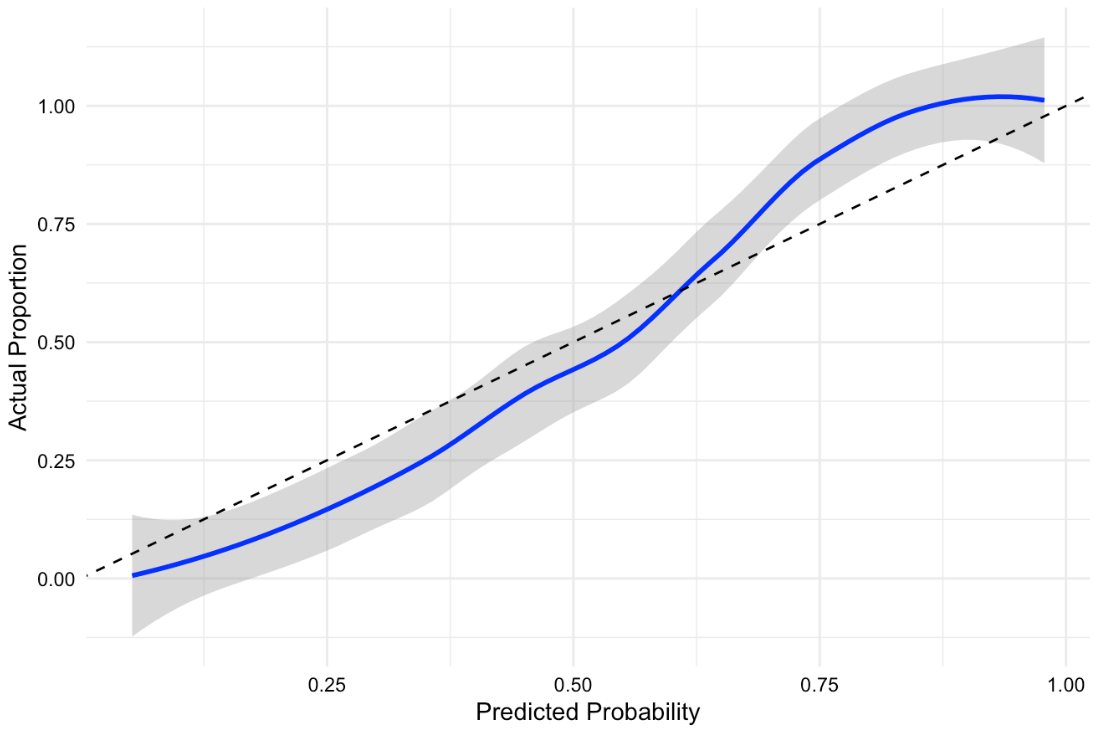

Supplementary Figure 3. Calibration Plot of the RF Model in the External Validation Cohort.

The calibration plot assesses the agreement between predicted probabilities and observed proportions of HCA (stage ≥ II). The diagonal dotted line (identity line) represents perfect calibration. The blue solid line, red dashed line, and green dotted line correspond to different calibration estimates (e.g., flexible calibration curves, grouped estimates, or adjusted estimates) derived from the validation cohort. The close proximity of these curves to the identity line across most predicted probability levels (especially in the mid to high ranges) indicates good calibration of the model.
