# Supplementary material for: Machine learning for prediction of histologic chorioamnionitis (stage ≥II) in parturients receiving labor analgesia: a retrospective multicentre cohort study
Source: Front Med (Lausanne). 2026 Jun 17;13:1841139. doi: 10.3389/fmed.2026.1841139 (PMC13318988; doi:10.3389/fmed.2026.1841139)
Supplement: Supplementary file 4 [file Table_1.docx]

**Supplementary Table 1.** Hyperparameter search ranges and final selected values for LR, RF, and XGBoost models.

| **Model** | **Hyperparameters (Search Range)** | **Final Selected Values** |
| --- | --- | --- |
| LR | penalty {l1, l2}; C (0.01–10, log) | l2; 0.5 |
| RF | n_estimators (100–500); mtry (1–6); max_depth (3–20) | 200; 2; 10 |
| XGBoost | n_estimators (50–300); max_depth (3–10);  learning_rate (0.01–0.3); subsample (0.5–1.0) | 150; 6; 0.1; 0.8 |

**LR, logistic regression; RF, random forest; XGBoost, extreme gradient boosting; C, inverse regularization strength; n_estimators, number of trees; mtry, number of variables randomly sampled as candidates at each split; max_depth, maximum depth of a tree; learning_rate, step size shrinkage; subsample, subsample ratio of training instances.**
