# Supplementary material for: Machine learning for prediction of histologic chorioamnionitis (stage ≥II) in parturients receiving labor analgesia: a retrospective multicentre cohort study
Source: Front Med (Lausanne). 2026 Jun 17;13:1841139. doi: 10.3389/fmed.2026.1841139 (PMC13318988; doi:10.3389/fmed.2026.1841139)
Supplement: Supplementary file 5 [file Table_2.docx]

**Supplementary Table 2.** Highly correlated variable pairs among candidate continuous variables (correlation coefficient |r| > 0.6).

| **Characteristics** | **Characteristics** | **Correlation coefficient (r)** |
| --- | --- | --- |
| WBC | NEUT | 0.987 |
| Mono | M% | 0.778 |
| NLR | PLR | 0.736 |
| NLR | MLR | 0.694 |
| NLR | LYM | -0.687 |
| PLR | LYM | -0.632 |
| RBC | Hb | 0.617 |

**Abbreviations:** WBC, white blood cell count; NEUT, neutrophil count; Hb, hemoglobin; LYM, lymphocyte count; Mono, monocyte count; NLR, neutrophil-to-lymphocyte ratio; MLR, monocyte-to-lymphocyte ratio; PLR, platelet-to-lymphocyte ratio; M%, monocyte percentage.
