# Supplementary material for: Machine learning for prediction of histologic chorioamnionitis (stage ≥II) in parturients receiving labor analgesia: a retrospective multicentre cohort study
Source: Front Med (Lausanne). 2026 Jun 17;13:1841139. doi: 10.3389/fmed.2026.1841139 (PMC13318988; doi:10.3389/fmed.2026.1841139)
Supplement: Supplementary file 6 [file Table_3.docx]

**Supplementary Table 3. Selection of variables among highly correlated pairs based on point-biserial correlation with the outcome (HCA stage ≥ II).**

| **Correlated Pair** | **Characteristics** | **Point-Biserial r with Outcome** | ***P* Value** | **Retained Variable** |
| --- | --- | --- | --- | --- |
| WBC vs NEUT | WBC | 0.009 | 0.7439 | NEUT |
|  | NEUT | 0.019 | 0.4773 |  |
| Mono vs M% | Mono | 0.028 | 0.4145 | Mono |
|  | M% | 0.017 | 0.5209 |  |
| NLR vs PLR | NLR | 0.043 | 0.0992 | NLR |
|  | PLR | -0.024 | 0.3505 |  |
| NLR vs MLR | NLR | 0.043 | 0.0992 | MLR |
|  | MLR | 0.076 | 0.0035 |  |
| NLR vs LYM | NLR | 0.043 | 0.0992 | LYM |
|  | LYM | -0.081 | 0.0019 |  |
| PLR vs LYM | PLR | -0.024 | 0.3505 | LYM |
|  | LYM | -0.081 | 0.0019 |  |
| RBC vs Hb | RBC | -0.015 | 0.5742 | Hb |
|  | Hb | -0.053 | 0.0431 |  |

**Abbreviations:** WBC, white blood cell count; NEUT, neutrophil count; Hb, hemoglobin; LYM, lymphocyte count; Mono, monocyte count; NLR, neutrophil-to-lymphocyte ratio; MLR, monocyte-to-lymphocyte ratio; PLR, platelet-to-lymphocyte ratio; M%, monocyte percentage.

**Final continuous variables retained for modeling: NEUT , Mono, NLR, MLR, LYM count, Hb.**
