# Supplementary material for: Machine learning for prediction of histologic chorioamnionitis (stage ≥II) in parturients receiving labor analgesia: a retrospective multicentre cohort study
Source: Front Med (Lausanne). 2026 Jun 17;13:1841139. doi: 10.3389/fmed.2026.1841139 (PMC13318988; doi:10.3389/fmed.2026.1841139)
Supplement: Supplementary file 7 [file Table_4.docx]

**Supplementary Table 4.** Brier scores of the three models on the internal validation set

| **Model** | **Brier Score** |
| --- | --- |
| LR | 0.160 |
| RF | 0.081 |
| XGBoost | 0.089 |

**Abbreviations: LR,** logistic regression; RF, random forest; XGBoost, extreme gradient boosting.
