# Supplementary material for: Machine learning for prediction of histologic chorioamnionitis (stage ≥II) in parturients receiving labor analgesia: a retrospective multicentre cohort study
Source: Front Med (Lausanne). 2026 Jun 17;13:1841139. doi: 10.3389/fmed.2026.1841139 (PMC13318988; doi:10.3389/fmed.2026.1841139)
Supplement: Supplementary file 8 [file Table_5.docx]

**Supplementary Table 5.** Comparisons of the model performance with variable exclusion

| **Characteristics** | **AUC (95% CI)** |
| --- | --- |
| **Exclusion: Maximum temperature** |  |
| Test set | 0.935 (0.912–0.959) |
| validation cohort | 0.781 (0.742–0.820) |
| **Exclusion: Gestational age** |  |
| Test set | 0.931 (0.909-0.954) |
| validation cohort | 0.737 (0.692-0.782) |
| **Exclusion: BMI** |  |
| Test set | 0.944 (0.925-0.963) |
| validation cohort | 0.759 (0.717-0.802) |
| **Exclusion: CRP** |  |
| Test set | 0.941 (0.918–0.963) |
| validation cohort | 0.989 (0.978–0.999) |
| **Exclusion: Meconium-stained amniotic fluid** |  |
| Test set | 0.946 (0.928–0.965) |
| validation cohort | 0.816 (0.780–0.852) |
| **Exclusion: PLT** |  |
| Test set | 0.949 (0.931–0.967) |
| validation cohort | 0.807 (0.770–0.844) |

Abbreviations: BMI, Body mass index; PLT, platelet count; CRP, C-reactive protein.
